# Dyslipidemia and associated factors among women using hormonal contraceptives in Harar town, Eastern Ethiopia

**DOI:** 10.1186/s13104-019-4148-9

**Published:** 2019-03-04

**Authors:** Berhanu Sufa, Gemeda Abebe, Waqtola Cheneke

**Affiliations:** 1 Department of Medical Laboratory Science, Harar Health Science College, Harar, Ethiopia; 2 School of Medical Laboratory Sciences, Faculty of Health Sciences, Institute of Health, Jimma University, Jimma, Ethiopia

**Keywords:** Prevalence, Dyslipidemia, Hormonal contraceptives, Risk factor, Ethiopia

## Abstract

**Objective:**

Dyslipidemia is abnormal amount of lipid in blood. Hormonal contraceptives affect lipid metabolism and can enhance the risk of vascular disease like atherosclerosis. In Harar, among contraceptive users, biochemical changes follow up is almost none and magnitude of dyslipidemia is not known. Therefore this study is designed to determine prevalence of dyslipidemia and its predisposing factors. Accordingly, cross-sectional study was conducted from April to June 2014 among hormonal contraceptive users from three health centers and one hospital. Socio-demographic data, anthropometric measurements, and blood biochemical tests were performed for every participant. Descriptive statistics and logistic regression analysis with 95% confidence interval using SPSS was used.

**Result:**

Totally 365 participants were included and the prevalence of dyslipidemia was 34.8%. The mean levels ± standard deviation of total cholesterol, low density lipoprotein (LDL), high density lipoprotein (HDL), the total cholesterol to HDL ratio, and triglyceride were 186 ± 27 mg/dl, 121 ± 31 mg/dl, 45.21 ± 7.7 mg/dl, 4.44, and 108 ± 3.45 mg/dl, respectively. Age, fasting blood sugar, drinking coffee twice and eating no vegetables 4 times/week were identified as predictors of dyslipidemia. In conclusion, hormonal contraceptive users of Harar have high rate of dyslipidemia. This result emphasizes the urgent need for a public health strategy for prevention, early detection, and treatment of dyslipidemia.

**Electronic supplementary material:**

The online version of this article (10.1186/s13104-019-4148-9) contains supplementary material, which is available to authorized users.

## Introduction

Dyslipidemia is a condition that occurs because of abnormalities in the plasma lipids such as elevated plasma total cholesterol (TC), elevated low-density lipoprotein cholesterol (LDL-C), elevated triglycerides (TG) and reduced high-density lipoprotein cholesterol (HDL-C) levels, occurring singly or in combinations [1]. Together with other cardiovascular risk factors dyslipidemia may influence the progression of atherosclerosis [2]. Cardiovascular disease (CVD) is the leading cause of death in women and the third leading cause of death among women of reproductive age. During 2005 through 2008, 11% of women aged 20 to 44 years had dyslipidemia [3, 4]. Hormonal contraceptives affect cardiac function, blood pressure (BP), fat and carbohydrate metabolism [5]. The resultant effect of contraceptive hormones on lipid metabolism depends on the type and dose of the compounds, the route of administration, and the duration of treatment [6]. Prolonged use of hormonal contraceptives by females during their reproductive age can induce metabolic changes that may contribute to an increased risk of coronary heart [7]. It is now generally accepted that both elevated levels of non-HDL-C and low HDL-C concentrations may promote the development of atherosclerosis [8]. As the resultant effect of combined oral contraceptives (COC) on the formation of dyslipidemia depends on different factors and dyslipidemia promotes development of atherosclerosis, determining magnitude of dyslipidemia and other cardiovascular risk factors among women using hormonal contraceptive helps to aware health care workers to use options for its management. However, there is no study for determination of dyslipidemia and cardiovascular risk factors for women using hormonal contraceptives in all health facilities in Harar town. Hence, the objective of this study is to determine the prevalence of dyslipidemia and associated factor among women using hormonal contraceptives.

## Main text

### Methods

#### Study setting and participants

A cross sectional study was conducted from April to June, 2014 in Harar Eastern Ethiopia, which was selected randomly and is 515 km away from Addis Ababa. Sample size was determined using single population proportion formula and then corrected by finite population correction formula to include a total of 365 hormonal contraceptive users having complete recorded data, satisfied the inclusion criteria and volunteer to participate in the study. To include participants randomly from the town, we stratified the town into three areas and randomly selected at least 1 public health institution from every area. Participants were included consecutively from 3 health facilities (2 health centers and 1 hospital) in the town till the determined sample size was achieved. The 3 facilities were selected randomly. The 365 sample size was divided proportionately among the 2 health centers and the 1 hospital as 1:1:1.65 ratios, respectively. Hormonal contraceptive users with the current history of pregnancy; those who have history of hypertension, diabetes, HIV/AIDS, mental disorders and those who were on lipid lowering medications were excluded from the study.

#### Data collection and laboratory testing

Socio demographic and clinical data were collected using structured questionnaire and checklist. Each consented participants were informed about the importance of obtaining overnight fasting blood specimen and advised to come early in the morning by the next day; then about 10 ml of venous blood was collected from each into clean serum separator test tubes and allowed to clot for 30 min. After retracting the clot the samples were centrifuged at 3000 rpm’s for 10 min and 5 ml pure serum samples were transferred to nunc tubes and analyzed for fasting blood sugar (FBS), TC, TG, and LDL-C using Mindray BS-120 chemistry analyzer. HDL-C was calculated in serum specimens having a TG value < 400 mg/dl by reverse method using Friedewald formula.

Standard operating procedures and manufacturer instructions were strictly followed throughout the procedures. Quality control run was undertaken for all laboratory tests in this study.

#### Statistical analysis

Data was edited, cleaned, coded and entered using Epidata and then exported to SPSS for analysis. Mean, standard deviation and frequency of quantitative variables were calculated. Bivariate and multivariate logistic regression analysis was calculated with 95% confidence interval (CI) to evaluate the possible association of the variables and p-value of less than 0.05 was considered as statistically significant.

### Results

#### Demographic and anthropometric data

A total of 365 consented women were enrolled in this study with overall response rate of 100%. The mean plus or minus standard deviation (± SD) age of the participants was 30.1 (± 6.7) years. Majority, 163 (44.7%), of the participants were 20–29 years old. The mean height and weight of the subjects were 161 (± 0.06) cm and 57.9 (± 6.8) kg, respectively. Majority, 187 (51.2%), of the participants were Muslim by religion and Oromo by ethnicity [162 (44.4%)]. Educationally, majority of them, 206 (56.4%), attended above secondary school; occupationally 157 (43%) of them were housewives and 307 (84.1%) were married. The mean monthly income of the family was 1786.6 Ethiopian birr (ETB) (Table 1).

**Table 1:** Associations of socio-demographic factors with dyslipidemia women using hormonal contraceptives by using binary logistic regression in Harar town, April–June; 2014

	Characteristics	Dyslipidemia			COR (95% CI)	p-value
		No (N/%)	Yes (N/%)	Total		
Age	< 20	12 (92.3)	1 (7.7)	13	1	
	20–29	73 (47.7)	80 (52.3)	163	2.60 (0.325, 20.769)	0.368
	30–39	134 (82.2)	29 (17.8)	153	13.15 (1.669, 103.647)	0.014
	≥ 40	19 (52.8)	17 (47.2)	36	10.74 (1.260, 91.47)	0.030
Marital status	Widowed	2 (66.7)	1 (33.3)	3	1	
	Married	199 (64.8)	108 (35.2)	307	1.085 (0.097, 12.11)	0.947
	Single/divorced	37 (67.3)	18 (32.7)	55	1.29 (0.101, 16.2)	0.64
Ethnicity	Gurage	22 (62.9)	13 (37.1)	35	1	
	Oromo	104 (64.2)	58 (35.8)	162	0.944 (0.443, 2.012)	0.88
	Amhara	68 (71.6)	27 (28.4)	95	0.0.67 (0.297, 1.52)	0.341
	Harari	44 (60.3)	29 (39.7)	73	1.115 (0.49, 2.56)	0.191
Education	Illiterate	52 (66.7)	25 (33.3)	77	1	
	Primary school	54 (66.7)	27 (33.3)	81	1.000 (0.517, 1.93)	1.000
	Secondary school	74 (72.5)	28 (27.5)	102	0.757 (0.39, 1.44)	0.394
	≥ Higher education	58 (55.8)	46 (44.2)	104	1.59 (0.86, 2.92)	0.797
Religion	Orthodox	85 (67.5)	41 (32.5)	126	1	
	Muslim	119 (63.6)	68 (36.4)	187	1.185 (0.74, 1.91)	0.490
	Protestant	34 (65.4)	18 (34.6)	52	1.10 (0.56, 2.17)	0.789
Income	≤ 1500	135 (69.2)	60 (30.8)	195	1	
	> 1500	103 (60.6)	67 (39.4)	170	1.464 (0.95, 2.256)	0.084
Occupation	House wife	105 (66.9)	52 (33.1)	157	1	
	Merchant	45 (67.2)	22 (32.8)	67	0.987 (0.537, 1.814)	0.967
	Employed	85 (61.6)	53 (38.4)	138	1.26 (0.78, 2.030)	0.345
	Others	3 (100)	0 (0)	3	0.000	0.999

*COR* crude odds ratio

#### Clinical and laboratory data

The mean (± SD) levels of TC, LDL-C, HDL-C, TG, and the TC/HDL-C ratio of the study subjects were 186 (± 27) mg/dl, 121 (± 31) mg/dl, 45.21 (± 7.7) mg/dl, 108 (± 3.45) mg/dl, and 4.44, respectively. The age-adjusted mean (± SD) levels of the lipid profiles are presented in tables (Additional file 1: Table S1). Moreover their mean (± SD) in comparison with types and duration of contraceptives used are presented in tables (Additional file 2: Table S2 and Additional file 3: Table S3).

A total of 30 (8.2%) participants had a FBS > 126 mg/dl and 73 (20%) had body mass index (BMI) > 25 kg/m^2^. Only 12 (3.3%) participants had systolic blood pressure (SBP) > 140 mmHg and 23 (6.3%) had diastolic blood pressure (DBP) > 90 mmHg. Moreover, 94 (25.8%) had a waist-to-hip ratio > 0.85; 16 (4.4%), 21 (5.8%), and 11 (3%) had family history of hypertension, of diabetes mellitus and of obesity, respectively. Likewise, 203 (55.6%) drink coffee; of which 163 (44.7%) drink at least once a day and 40 (11%) drink more than twice a day. Similarly 186 (51%) make physical exercise for at least 30 min a day of which 176 (48.2%) perform < 3 times a week and 10 (2.7%) > 4 times a week. Furthermore, 17 (4.4%) participants drink alcohol and 69 (18.9%) chew khat at least once a week (Table 2).

**Table 2:** Associations of cardiovascular and life style factors with dyslipidemia among contraceptive user women by using binary logistic regression in Harar town, 2014

	Variables	Dyslipidemia			COR (95% CI)	p-value
		No (N/%)	Yes (N/%)	Total		
SBP (mmHg)	≤ 139	236 (66.9)	117 (33.1)	353	1	
	≥ 140	2 (16.7)	10 (83.3)	12	10.085 (2.175, 46.78)	0.003
Diastolic blood pressure (mmHg)	≤ 89	233 (68.1)	109 (31.9)	342	1	
	≥ 90	5 (21.7)	18 (78.3)	23	7.695 (2.78, 21.27)	0.000
Body mass index (kg/m^2^)	< 18.50	8 (88.9)	1 (11.1)	9	1	
	18.50–24.9	217 (76.7)	66 (23.3)	283	2.433 (0.299, 19.811)	0.406
	25–29.99	13 (18.6)	57 (81.4)	70	35.077 (4.028, 305.49)	0.000
	≥ 30	0 (0)	3 (100)	3	0	0.999
Waist to hip ratio	≤ 0.85	198 (73.1)	73 (26.9)	271	1	
	> 0.85	40 (42.6)	54 (57.4)	94	3.662 (2.245, 5.97)	0.000
Fasting blood sugar (mg/dl)	< 110	222 (88.1)	30 (11.9)	252	1	
	110–125	13 (15.7)	70 (84.3)	83	39.85 (19.706, 80.57)	0.000
	≥ 126	3 (10)	27 (90)	30	66.6 (19.038, 232.98)	0.000
Family history of diabetes mellitus	No	233 (67.7)	111 (32.3)	344	1	
	Yes	5 (23.8)	16 (76.2)	21	6.717 (2.400, 18.802)	0.000
Family history of hypertension	No	234 (67)	115 (33.0)	349	1	
	Yes	4 (25)	12 (75)	16	6.104 (1.926, 19.344)	0.002
Family history of obesity	No	234 (66.1)	120 (33.9)	354	1	
	Yes	4 (36.4)	7 (63.6)	11	0.293 (0.084, 1.021)	0.054
Coffee	No	119 (73.5)	43 (26.5)	162	1	
	< 2 times/day	108 (66.3)	55 (33.7)	163	1.409 (0.875, 2.270)	0.158
	≥ 2 times/day	11 (27.5)	29 (72.5)	40	7.296 (3.355, 15.86)	0.000
Physical exercise	> 3 times/week	8 (80)	2 (20)	10	1	
	≤ 3 times/week	147 (83.5)	29 (16.5)	176	0.171 (0.104, 0.280)	0.057
	No	83 (46.4)	96 (53.6)	179	0.216 (0.045, 1.046)	0.000
Smoking	No	234 (65)	126 (35)	360	1	
	Yes	4 (80)	1 (20)	5	2.154 (0.238, 19.477)	0.495
Alcohol use	No	224 (64.4)	124 (35.6)	348	1	
	Yes	14 (82.4)	3 (17.6)	17	2.583 (0.728, 9.163)	0.142
Chew khat	No	197 (66.6)	99 (33.4)	296	1	
	Yes	41 (59.4)	28 (40.6)	69	0.736 (0.430, 1.260)	0.264
Parity	Nulliparous	40 (83.3)	8 (16.7)	48	1	
	1	73 (76)	23 (24)	96	1.575 (0.646, 3.844)	0.318
	2	57 (53.3)	50 (46.7)	107	4.386 (1.877, 10.25)	0.001
	3	36 (63.2)	21 (36.8)	57	2.917 (1.150, 7.396)	0.024
	4	32 (56.1)	25 (43.9)	57	3.906 (1.554, 9.821)	0.004
Types of hormonal contraceptives	Jedalle/norplant	14 (60.9)	9 (39.1)	23	1	
	Injectable	127 (59.9)	85 (40.1)	212	1.041 (0.431, 2.513)	0.429
	OCP	38 (71.7)	15 (28.3)	53	0.614 (0.219, 1.718)	0.353
	Implanon	59 (76.6)	18 (23.4)	77	0.475 (0.176, 1.277)	0.140
Duration (in months)	6–18	127 (75.6)	41 (24.4)	168	1	
	18–30	63 (71.6)	25 (28.4)	88	1.229 (0.687, 2.199)	0.487
	30–42	31 (56.4)	24 (43.6)	55	2.398 (1.266, 4.542)	0.007
	> 42	17 (31.5)	37 (68.5)	54	6.742 (3.437, 13.22)	0.000
Eat meat/week	No	37 (78.7)	10 (21.3)	47	1	
	≤ 4 times	196 (64.9)	106 (35.1)	302	2.001 (0.957, 4.183)	0.065
	> 4 times	5 (31.2)	11 (68.8)	16	8.140 (2.293, 28.901)	0.001
Eat veg./week	> 4 times/week	70 (71.4)	28 (28.6)	98	1	
	≤ 4 times	165 (63.2)	96 (36.8)	261	2.500 (0.476, 13.138)	0.250
	No	3 (50)	3 (50)	6	1.455 (0.878, 2.411)	0.126
Use milk	No	56 (72.7)	21 (27.3)	77	1	
	≤ 4 times	159 (71)	65 (29)	224	0.090 (0.611, 1.944)	0.77
	> 4 times	23 (35.9)	41 (64.1)	64	4.754 (2.32, 9.724)	0.000

*mmHg* millimeter mercury, *kg/m*^*2*^ kilogram per meter square, *mg/dl* milligram per deciliter

#### Prevalence of dyslipidemia

Dyslipidemia or abnormal values of TC, TG, LDL, HDL and TC/HDL ratio were identified, with overall prevalence of 34.8% (Additional file 4: Figure S1). Socio-demographic variables such as age, occupation, income, religion, marital status and educational status were taken as study variables. The presence of dyslipidemia was statistically different among age categories. However, there was no difference among religion, ethnicity, marital status, educational status and occupation (Table 1). Additionally, the presence of dyslipidemia was assessed based on BP, BMI, waist circumference, waist-to-hip ratio (WHR), FBS, life style, feeding habits, family history of selected medical condition, parity and type and duration of hormonal contraceptives. Women with dyslipidemia were 10 times likely to have SBP > 140 mmHg (Odds ratio (OR) = 10.085; 95% CI 2.175–46.78, p = 0.003) and 8 times likely to have DBP > 90 mmHg (OR = 7.695; 95% CI 2.78–21.27, p = 0.000) as compared to their counterparts. However, there was no difference in dyslipidemia between types of contraceptives, drinking alcohol and cigarette smoking (Table 2). The proportion of women having dyslipidemia in comparison with the type and duration of use of contraceptive are presented in table (Additional file 5: Table S4). The percentage of dyslipidemia in respect to individual biochemical parameters of the lipid profiles are displayed in table (Additional file 6: Table S5).

#### Predictors of dyslipidemia

The significant predictors of dyslipidemia in this study were age, FBS, not eating vegetables and drinking coffee twice. Women who do not eat vegetables were about 3 times more likely to develop dyslipidemia (adjusted odds ratio (AOR) = 3.14; 95% CI 1.27–7.77, p = 0.013) and those who drink coffee more than 2 times per day were about 5 times more likely to develop dyslipidemia (AOR = 4.810; 95% CI 1.23–18.76, p = 0.024) (Table 3).

**Table 3:** Multivariate analysis of independent risk factors for dyslipidemia in women using contraceptive hormones; in Harar town, April–June 2014

	Variables	Dyslipidemia			AOR (95% CI)	p-value
		No (N/%)	Yes (N/%)	Total		
Age (years)	< 20	12 (92.3)	1 (7.7)	13	1	
	20–29	73 (47.7)	80 (52.8)	153	33.43 (1.23, 45.2)	0.065
	30–39	134 (82.2)	29 (17.8)	163	44.45 (1.104, 18)	0.044
	≥ 40	19 (52.8)	17 (47.2)	36	28.05 (1.44, 30.9)	0.098
FBS (mg/dl)	≤ 110	222 (88.1)	30 (11.9)	252	1	
	110–126	13 (15.7)	70 (84.3)	83	35.95 (14.35,89.90)	0.000
	> 126	3 (10)	27 (90)	30	35.75 (8.66, 147.50)	0.000
Eat vegetables	> 4 times/week	70 (71.4)	28 (28.6)	98	1	
	No	3 (50)	3 (50)	6	3.14 (1.27,7.77)	0.013
	≤ 4 times	196 (64.9)	106 (35.1)	302	2.09 (0.87, 5.23)	0.070
Drink coffee	No	119 (73.5)	43 (26.5)	162	1	
	≥ 2 times/day	11 (27.5)	29 (72.5)	40	4.810 (1.23, 18.76)	0.024

*AOR* adjusted odds ratio, *FBS* fasting blood sugar, *mg/dl* milligram per deciliter, *CI* confidence interval

### Discussion

Dyslipidemia is a common public health problem in developing countries, the prevalence of which is rising steadily [9]. To the best of our knowledge this study is the first to examine prevalence of dyslipidemia and associated factors in Ethiopia. In this study the prevalence of dyslipidemia was found to be 34.8% which is lower than finding in Iran (63.4%) [9].

The predictors of dyslipidemia were age > 30 years, FBS > 110 mg/dl, coffee drinking > 2 times a day and not eating vegetables. The result for not eating vegetables agrees with the study conducted in Framingham [10] but literature on the effect of coffee on serum lipids in women using hormonal contraceptive is limited.

The mean levels of lipid profile are different from finding in Iraq [11]. This difference might be due to the socio-demographic and socioeconomic status of the study subjects.

The prevalence of hypercholesterolemia (TC ≥ 200 mg/dl) was 33.7% and the mean TC level (186 ± 27 mg/dl) of this study is comparable with that in Brazil [12] and Germany [13] but lower than Bangladesh, Dhaka [14] and higher than Iraq [11]. Significantly increased TC might be due to differences in socioeconomic, life style, methodology and duration and type of contraceptives.

The prevalence of hyperlipidemia (LDL-C ≥ 130 mg/dl) is found to be 34.8% and the mean LDL-C (121 ± 31 mg/dl) is lower than study in Bangladesh, Dhaka [14], but higher than Brazil [12], Germany [13] and Iraq [11]. This variation might be due to differential distribution in risk factors.

The prevalence of hypolipidemia (HDL-C < 40 mg/dl) was 28.2%. The alteration in the reference value, as to the new recommendations [15] made comparison difficult with the previous studies. The mean HDL-C (45.21 ± 7.7 mg/dl) is lower compared with study in Brazil [12], Germany [13] and Iraq [11] but higher than Bangladesh, Dhaka [14].

The prevalence of hypertriglyceridemia was found to be 17.0% which is comparable with studies in developing countries including Africa where its level ranges from 15 to 30% [16]. The mean TG (107 ± 34 mg/dl) is comparable with finding in Bangladesh, Dhaka [14] but, lower than Basra, Iraq [11] and higher than Pakistan [7]. The decreased prevalence of hypertriglyceridemia in this study might be related to hormonal contraceptive induced increase in clearance of TG rather than synthesis and might be because of fasting.

The mean SBP (119.85 ± 9.5 mmHg) and DBP (77.96 ± 6.8 mmHg) are consistent with the study in Germany [13] but SBP is different from Basra, Iraq [11]. This variation might be due to difference of socio-demographic factors, genetic predisposition, dietary factors, and lack of physical activity.

The level of TC, TG and LDL-C has increased with duration of contraceptive intake but, HDL-C level is significantly decreased. This agrees with a study in Nigeria [17] but the LDL-C level is different from Basra; Iraq [11] in which it was decreased with duration of contraceptive use. Significant decrease (p < 0.05) of HDL-cholesterol was observed within the duration of different types of hormonal contraceptives [18–21].

Dyslipidemia was higher among groups with high educational level. This might be resulted from higher income, poor working conditions and poor nutritional habits.

The prevalence of dyslipidemia was significantly higher among employed 53 (38.4%) and house wives 52 (33.1%) which might be due to poor physical activity. The odds ratio for dyslipidemia is significantly greater in subjects with a family history of obesity, diabetes and hypertension. Cigarette smoking has no significant association with dyslipidemia which is similar with study conducted in Germany [13].

The prevalence of dyslipidemia has increased with parity, however; there was a fluctuating pattern in HDL-C level in between consecutive pregnancies which is similar to studies according to Mankuta et al. [21].

### Conclusion

This study revealed that dyslipidemia is high among the study subjects and age, FBS, drinking coffee twice and not eating vegetables were found to be the independent predictors of dyslipidemia. The finding of this study indicates the urgent need for public health strategy to prevent, detect, and treat the dyslipidemia. Public health measures should continue to emphasize on the importance of healthy feeding habits and screening for different metabolic disorders as well as provision and monitoring of the quality of family planning services. Moreover large scale longitudinal study is needed to determine the effect of hormonal contraceptives on serum lipid and lipoprotein levels. Thus, this study can be used as baseline information for further studies related to effect of hormonal contraceptives on serum lipid levels.

## Limitations

As this study is cross sectional type, the long term effect could not be synthesized from the data. This study is also facility based but it would have been better if it was community based and the sample size was randomly selected from the community.

## Additional files

**Additional file 1: Table S1.** The age-adjusted mean ± SD values of plasma lipids and TC/HDL-C ratio in women using hormonal contraceptives in Harar by age groups, 2014.

**Additional file 2: Table S2.** The mean ± SD values of plasma lipids and TC/HDL-C ratio in women using hormonal contraceptives in Harar by types of contraceptive use, 2014.

**Additional file 3: Table S3.** The mean ± SD values of plasma lipids and TC/HDL-C ratio in women using hormonal contraceptives in Harar by duration of contraceptive use in months, 2014.

**Additional file 4: Figure S1.** Prevalence of dyslipidemia among women using hormonal contraceptives in Harar town, 2014.

**Additional file 5: Table S4.** Dyslipidemia versus types and duration of hormonal contraceptive users in Harar town, April–June; 2014.

**Additional file 6: Table S5.** Distribution of dyslipidemia with respect to individual biochemical parameters among women using hormonal contraceptives in Harar town; April–June; 2014.

## Abbreviations

AIDS: acquired immunodeficiency syndrome
AOR: adjusted odds ratio
BMI: body mass index
BP: blood pressure
CI: confidence interval
COC: combined oral contraceptive
CVD: cardiovascular disease
DBP: diastolic blood pressure
HDL: high density lipoprotein
HIV: human immunodeficiency virus
LDL: low density lipoprotein
mg/dl: milligram per deciliter
mmHg: millimeter mercury
OR: odds ratio
SBP: systolic blood pressure
SD: standard deviation
TC: total cholesterol
TC/HDL: total cholesterol: high density lipoprotein
TG: Triglyceride
WHR: waist to hip ratio
