# Supplementary material for: Dyslipidemia and associated factors among women using hormonal contraceptives in Harar town, Eastern Ethiopia
Source: BMC Res Notes. 2019 Mar 4;12:120. doi: 10.1186/s13104-019-4148-9 (PMC6399905; doi:10.1186/s13104-019-4148-9)
Supplement: Supplementary file 1 — Additional file 1: Table S1. The age-adjusted mean ± SD values of plasma lipids and TC/HDL-C ratio in women using hormonal contraceptives in Harar by age groups, 2014. [file 13104_2019_4148_MOESM1_ESM.doc]

**Table S1**: The age-adjusted mean ± SD values of plasma lipids and TC/HDL-C ratio in women using hormonal contraceptives in Harar by age groups, 2014.

|  | ***TC (mg/dl)*** | ***LDL-C (mg/dl)*** | ***HDL-C (mg/dl)*** | ***TC/HDL-C Ratio*** | ***TG (mg/dl)*** |
| --- | --- | --- | --- | --- | --- |
| **< 20** | 166.3 ±7.6 | 101.3 ± 7.3 | 48.7 ± 1.6 | 3.54 ± 0.33 | 84.5 ± 8.90 |
| **20–29** | 173.8 ±2.1 | 107.6 ± 2.2 | 48.4 ± 0.5 | 3.73 ± 0.10 | 92.3 ± 2.50 |
| **30–39** | 197.7 ±2.2 | 133.2 ± 2.5 | 42.00± 0.6 | 5.15 ± 0.15 | 121.2 ± 2.54 |
| **40–49** | 202.2 ±3.2 | 136.9 ± 4.5 | 43.0 ± 1.2 | 4.94 ± 0.25 | 126.6 ± 3.64 |
| **Total** | 184.95±3.8 | 119.75± 4.1 | 45.5± 0.98 | 4.34± 0.21 | 106.15±4.40 |
