# Supplementary material for: Dyslipidemia and associated factors among women using hormonal contraceptives in Harar town, Eastern Ethiopia
Source: BMC Res Notes. 2019 Mar 4;12:120. doi: 10.1186/s13104-019-4148-9 (PMC6399905; doi:10.1186/s13104-019-4148-9)
Supplement: Supplementary file 2 — Additional file 2: Table S2. The mean ± SD values of plasma lipids and TC/HDL-C ratio in women using hormonal contraceptives in Harar by types of contraceptive use, 2014. [file 13104_2019_4148_MOESM2_ESM.doc]

**Table S1**: The mean ± SD values of plasma lipids and TC/HDL-C ratio in women using hormonal contraceptives in Harar by types of contraceptive use, 2014

| **Types of contraceptives** | **TC(mg/dl)** | **LDL_C**  **(mg/dl)** | **HDL_C**  **(mg/dl)** | **TC/HDL-C ratio** | **TG(mg/dl)** |
| --- | --- | --- | --- | --- | --- |
| Injectable | 189.35±2.0 | 124.13±2.1 | 44.12±.5 | 4.66±.1 | 111.56±2.3 |
| OCP | 181.981±3.6 | 115.87±3.7 | 46.62±.9 | 4.10±.2 | 102.83±4.3 |
| Implanon | 179.364±3.5 | 115.40±4.1 | 47.24±.8 | 4.07±.2 | 99.03±4.0 |
| Norplant/Jadelle | 187.30±6.1 | 122.65±6.3 | 45.23±1.7 | 4.46±.3 | 109.87±7.3 |
