# Supplementary material for: Dyslipidemia and associated factors among women using hormonal contraceptives in Harar town, Eastern Ethiopia
Source: BMC Res Notes. 2019 Mar 4;12:120. doi: 10.1186/s13104-019-4148-9 (PMC6399905; doi:10.1186/s13104-019-4148-9)
Supplement: Supplementary file 3 — Additional file 3: Table S3. The mean ± SD values of plasma lipids and TC/HDL-C ratio in women using hormonal contraceptives in Harar by duration of contraceptive use in months, 2014. [file 13104_2019_4148_MOESM3_ESM.doc]

Table S1: The mean ± SD values of plasma lipids and TC/HDL-C ratio in women using hormonal contraceptives in Harar by duration of contraceptive use in months, 2014

| **Duration of COC**  **use in months** | **TC(mg/dl)** | **LDL_C**  **(mg/dl)** | **HDL_C**  **(mg/dl)** | **TC/HDL-C ratio** | **TG(mg/dl)** |
| --- | --- | --- | --- | --- | --- |
| **6-18** | 179.5±1.97 | 113.4±1.99 | 46.8±.51 | 4.0±.10 | 100.1±2.35 |
| **18-30** | 181.6±3.08 | 116.6±3.26 | 46.5±.75 | 4.2±.18 | 102.4±3.58 |
| **30-42** | 191. 2±4.18 | 125.6±4.30 | 43.8±1.14 | 4.8± .24 | 113.3±5.03 |
| **>42** | 208.2±3.87 | 147.1±4.80 | 39.7±1.13 | 5.7± .25 | 133.1±4.46 |
