# Supplementary material for: Dyslipidemia and associated factors among women using hormonal contraceptives in Harar town, Eastern Ethiopia
Source: BMC Res Notes. 2019 Mar 4;12:120. doi: 10.1186/s13104-019-4148-9 (PMC6399905; doi:10.1186/s13104-019-4148-9)
Supplement: Supplementary file 4 — Additional file 4: Figure S1. Prevalence of dyslipidemia among women using hormonal contraceptives in Harar town, 2014. [file 13104_2019_4148_MOESM4_ESM.doc]

**Figure S1:** Prevalence of dyslipidemia among women using hormonal contraceptives in Harar town, 2014
