# Supplementary material for: Dyslipidemia and associated factors among women using hormonal contraceptives in Harar town, Eastern Ethiopia
Source: BMC Res Notes. 2019 Mar 4;12:120. doi: 10.1186/s13104-019-4148-9 (PMC6399905; doi:10.1186/s13104-019-4148-9)
Supplement: Supplementary file 5 — Additional file 5: Table S4. Dyslipidemia versus types and duration of hormonal contraceptive users in Harar town, April–June; 2014. [file 13104_2019_4148_MOESM5_ESM.doc]

**Table S1:** Dyslipidemia versus types and duration of hormonal contraceptive users in Harar town, April - June; 2014

| **Types and duration of Contraceptive use in months** | | **Dyslipidemia** | | |
| --- | --- | --- | --- | --- |
| **Types** | **Duration in months** | **No** | **Yes** | **Total** |
|  |  |  |  |  |
| **Injectable** | 6-18 | 70(72.2%) | 27(27.8%) | 97 |
|  | 18-30 | 42(71.2%) | 17(28.8%) | 59 |
|  | 30-42 | 8(33.3%) | 16(66.7%) | 24 |
|  | >42 | 7(21.9%) | 25(78.1%) | 32 |
| **OCP** | 6-18 | 30(78.9%) | 8(21.1%) | 38 |
|  | 18-30 | 4(80.0%) | 1(20.0%) | 5 |
|  | 30-42 | 3(50.0%) | 3(50.0%) | 6 |
|  | >42 | 1(25.0%) | 3(75.0%) | 4 |
| **Implanon** | 6-18 | 18(94.7%) | 1(5.3%) | 19 |
|  | 18-30 | 17(73.9%) | 6(26.1%) | 23 |
|  | 30-42 | 16(84.2%) | 3(15.8%) | 19 |
|  | >42 | 8(50.0%) | 8(50.0%) | 16 |
| **Jadelle /**  **Norplant** | 6-18 | 9(64.3%) | 5(35.7%) | 14 |
|  | 18-30 | 0(.0%) | 1(100.0%) | 1 |
|  | 30-42 | 4(66.7%) | 2(33.3%) | 6 |
|  | >42 | 1(50.0%) | 1(50.0%) | 2 |

OCP—Oral contraceptive pills
