# Supplementary material for: Dyslipidemia and associated factors among women using hormonal contraceptives in Harar town, Eastern Ethiopia
Source: BMC Res Notes. 2019 Mar 4;12:120. doi: 10.1186/s13104-019-4148-9 (PMC6399905; doi:10.1186/s13104-019-4148-9)
Supplement: Supplementary file 6 — Additional file 6: Table S5. Distribution of dyslipidemia with respect to individual biochemical parameters among women using hormonal contraceptives in Harar town; April–June; 2014. [file 13104_2019_4148_MOESM6_ESM.doc]

**Table S1: Distribution of dyslipidemia with respect to individual biochemical parameters among women using hormonal contraceptives in Harar town; April.-June; 2014**

| **Dyslipidemia** | **Frequency (%)**  N=365 |
| --- | --- |
| Total cholesterol | 123(33.7%) |
| Triglycerides | 62(17%) |
| LDL cholesterol | 127(34.8%) |
| HDL cholesterol | 103(28.2%) |
| TC/HDL ratio | 119(32.6%) |

*LDL-C--Low density Lipoprotein cholesterol; HDL-C--High density lipoprotein cholesterol*

*TC/HDL ratio ---- Total cholesterol to High density lipoprotein cholesterol ratio*
